# Teaching With Toons: Designing a Novel Blended-Learning Curriculum for Cognition and Dementia in Nursing Education

**DOI:** 10.1093/geroni/igaa057.008

**Published:** 2020-12-16

**Authors:** Bryan Brown, Gina Kang, Anna Schwartz, Richard Marottoli, Andrea Rink

**Affiliations:** 1 Yale School of Medicine, New Haven, Connecticut, United States; 2 Yale School of Public Health, Longmeadow, Massachusetts, United States; 3 Yale University, New Haven, Connecticut, United States

## Abstract

There is a shortage of learners completing nursing training and pursuing roles in geriatrics and dementia care, possibly caused by ageism, misconceptions, and personal experiences such as with family members, along with uncertainty and discomfort and providing care to this population. Using Kern’s six-steps of curriculum design, we set out to design a novel, blended-learning intervention to improve dementia education at a local nursing school. After reviewing the literature, local needs assessments were carried out in the form of stakeholder discussions and semi-structured interviews with a subset of nursing students. Interview results, themes from the literature, and incorporation of the current learning objectives in the existing curriculum, were integrated to create a new lesson plan, including a “flipped classroom” component using 2D vector animation, as well as animation-assisted, interactive, case-based lecture and discussion format. Media was designed through an iterative process including review of content outline, objectives, storyboards, and concept art by stakeholders and content experts throughout the design process. This novel approach to interdisciplinary, blended-learning curriculum design has the potential to improve nursing student attitudes and foundational knowledge about dementia and cognition.

